# Perception and Practice of Breastfeeding in Public in Poland

**DOI:** 10.34763/jmotherandchild.20212504.d-21-00020

**Published:** 2022-06-09

**Authors:** Justyna Grzyb, Łukasz Grzyb, Maria Wilińska

**Affiliations:** 1 Department of Neonatology, Prof. W. Orłowski Independent Public Clinical Hospital, Centre of Postgraduate Medical Education, Warsaw, Poland; 2 Polish Society of Actuaries, Warsaw, Poland; 3 Centre of Medical Postgraduate Education, Warsaw, Poland

**Keywords:** breastfeeding, public places, acceptance

## Abstract

**Background:**

The aim of the study was to get to know polish women’s opinions and experiences regarding breastfeeding in public.

**Material and methods:**

A one-time 11-question survey aimed at women during lactation or breastfeeding in the past who completed a paper questionnaire or online questionnaire on the website (www.laktacja.pl). The study was conducted electronically from 1 September 2019 to 31 March 2020 in Maternity and Neonatal Departments, primary health-care clinics in various Polish cities. Data from 700 questionnaires were statistically analysed with the use of Pearson’s chi-squared test of independency or Fisher’s exacts test when applicable (small cell counts).

**Results:**

90% of the surveyed women expressed the opinion that it should be possible to breastfeed in public, and 78% of women have had such an experience. Most often it was their own cars, a room for a mother and child, a hall or just a place available when there was a need to feed the child (e.g., a bench, cafe, toilet). About 10% of women faced criticism while breastfeeding in a public place, and 8.6% of women have never breastfed the child out of the house due to the lack of proper place and conditions, embarrassment and no sympathy from other people.

**Conclusion:**

Taking into account the benefits of long-term breastfeeding and the comfort of breastfeeding women, their children and the environment, it is necessary to create dedicated places for breastfeeding in public places.

## Introduction

Natural feeding is a commonly recommended method of feeding newborns, infants and young children [1]. The World Health Organization (WHO) recommends exclusive breastfeeding for the first 6 months of life and then breastfeeding until at least 2 years of age, along with expanding the diet [2]^.^ This model of nutrition ensures optimal growth and development of the child and has a positive effect on women's health (e.g. reducing the risk of metabolic syndrome, cardiovascular disease, type 2 diabetes, breast and ovarian cancer). [3, 4, 5, 6, 7, 8, 9, 10, 11, 12]. Breastfeeding is promoted both by the medical community, as well as by numerous NGOs and progressive social media [13].

Breast milk after the age of one is still an important component of the child’s diet, both in terms of nutrition, immunology and immunostimulation. The energy value of breast milk increases after the first year of lactation. In the second year of lactation, milk is characterised by a higher concentration of fats, total protein, lactoferrin, lysozyme and immunoglobulins, and a constant or increasing concentration of macronutrients and other bioactive factors [14, 15, 16, 17].

In Poland, almost all women start breastfeeding after childbirth. In the following months of a child's life, the share of mother's milk in the infant's diet drops drastically. At 6 months of age, only 4% of children in Poland are exclusively breastfed [18].

The needs of women who are breastfeeding for a long time are related to their professional activity, dealing with official matters and the need for social contacts [19]. Leaving the house with a child is not rarely associated with the need to feed the baby in a public place wherever the baby feels hungry.

A public place means “a place available to all or a certain segment of the public including, but not limited to, any place where the public is invited and is free to go upon special or implied invitation. It is specifically intended that public place shall include the interior of a vehicle if the vehicle is located at a public place” [20].

Among the many reasons for an early stopping breastfeeding is the reluctance of women to breastfeed in public due to the lack of social acceptance for nursing women, and additionally, it’s widely reported in literature [21, 22, 23, 24].

It seems that in some public places women are more consented to breastfeeding, but there are also others where it is still unwelcome. It may be related to the presence of rooms for mother and child in these places. It is also worth mentioning the lack of adequate space at work and time constraints. The possibility of using them may reduce the fear of an uncomfortable situation or a negative reaction from the environment [25,26].

Another factor that may have an impact on breastfeeding in public places is the presence or absence of protective legislation. In some countries in Northern Europe (Great Britain, Scotland, Netherlands, Norway, Sweden, Finland) and North America, breastfeeding in public places, including the workplace, is legally protected [27].

In Poland, there are no specific legal regulations concerning breastfeeding in public places [28].

The development of strategies to reduce challenges, enhance breastfeeding women's confidence and build a community where breastfeeding is the cultural norm should be prioritised by governments and organisations that promote breastfeeding [29].

The aim of the study was to find out about the experiences and feelings of women related to breastfeeding in the public places.

The following research hypotheses were formulated:

- Women believe that breastfeeding in public should be possible.
- Women are concerned that breastfeeding in public spaces may cause negative reactions from the environment.
- Due to concerns about the lack of social acceptance, women avoid breastfeeding in public.
- Women do not find adequate infrastructure in public spaces where they could breastfeed.
- Tolerance for seeing a woman breastfeeding in a public place depends on the size of the city of residence.

## Material and methods

The research method was a diagnostic survey using a paper and internet questionnaire.

A diagnostic survey, using paper and electronic questionnaires, was part of the work of the Committee for the Promotion of Breastfeeding at which the authors of the study work. The Bioethics Committee expressed a positive opinion on the commencement of the medical experiment (Resolution of the Bioethical Committee No. 24/2021 of April 14, 2021). The authors of the study used the data from the questionnaires to conduct the study with the approval of the Committee for the Promotion of Breastfeeding.

The questionnaire contained 11 single-choice closed questions and two semi-open questions that could be answered individually:

1. What do you think about breastfeeding in public places (gallery, train station, park, office)?- I can / I shouldn’t / I have no opinion;

2. Have you ever breastfed in a public place? – Yes / No;

3. If no, what was the reason? - There was no such need / There were no suitable conditions for this / I was ashamed / It is not well perceived by others / Other (semi-open question);

4. If yes, how often did you feed in a public place? – Occasionally / Sometimes / Often / Every time I've been in such a place;

5. If yes, was the place where you fed specially adapted to it? – Yes / No / Partly;

6. If yes, did you feel comfortable in the place where you breastfed? – Yes / No;

7. Have you ever been criticised for breastfeeding in public? – Yes / No;

8. If you had to breastfeed away from home, where was it most common? - In a room for a mother with a child / In the hotel lobby / In the toilet / In my car / Other (semi-open question);

9. Age - under 20 / between 20 and 30 / between 30 and 40 / over 40 years of age;

10. Size of the city of residence (size of the population) – small (< 20 000) / average (between 20 000 – 100 000) / large (over 100 000) / urban agglomeration (e.g. Warsaw, Katowice);

11. Level of education – primary / secondary / incomplete higher / higher education.

The data was collected in a paper questionnaire or an electronic questionnaire on the website (www.laktacja.pl) completed online [6].

The study was conducted from 1 September 2019 to 31 March 2020 among women in Maternity and Neonatal Departments and during visits to primary health-care clinics through a paper questionnaire. Some of the women completed the questionnaire disseminated on the website (www.laktacja.pl) and sent it electronically.

Non-probability sampling method was used in the study.

The research material consisted of the opinions of women who were breastfeeding or breastfeeding at present (N = 700).

Information from paper questionnaires was then entered into a common electronic database. The study was one-off.

Referring to semi-open questions (no open questions were used), the descriptive statistics, that is, numbers and percentages contained in the result tables and text of the article, encompass the most frequent answers to those semi-open survey questions. A quantitative content analysis was performed using Microsoft Excel.

The data were not analysed in the subgroups of breastfeeding women in the public space and women who have never breastfed in the public space mainly due to the aforementioned very large disproportion in the group size. Breastfeeding by the respondents in public was only a grouping (independent) variable in significance tests.

## Statistical analysis

Answers to the survey questions (categorical traits) were depicted as integral numbers and frequencies (percentages). Contingency tables, intersecting categorical variables, were provided with Pearson’s chi-squared test of independency, and Fisher’s exacts test when applicable (small cell counts). The statistical analyses were carried out by using IBM SPSS Statistics, version 28 (IBM Corporation, Armonk, NY, USA).

## Results

There were 705 responses collected. Five questionnaires were rejected due to incomplete data. Data from 700 questionnaires were analysed. The characteristics of the study group are presented in Table 1.

**Table 1:** Demographic characteristics of the surveyed women

Trait	N (%) *
Age (years)	
< 20	8 (1,1)
20-30	240 (34,3)
30-40	397 (56,7)
> 40	55 (7,9)

Size of the city of residence (size of the population)	
Urban agglomeration (e.g. Warsaw, Katowice)	234 (33,5)
Large city (over 100 000)	134 (19,2)
Average city (between 20 000 – 100 000)	206 (29,5)
Small city (< 20 000)	124 (17,8)

Level of education	
Primary education	10 (1,4)
Secondary education	138 (19,8)
Incomplete higher education	59 (8,5)
Higher education	490 (70,3)

(* Missing data were case-wise deleted.)

As shown in the table, the studied group of women differed in terms of age and place of residence. There are bigger differences in education. Almost three-fourths of the surveyed women have completed post-secondary education (Table 1).

Ninety percent of the women believe that breastfeeding in public should be possible. However, only three-quarters of respondents admitted that they had ever put a baby to the breast there. Only a quarter (25.5%) did it freely.

Unfortunately, only every tenth mother (7.8%) found a suitable place in the public space for breastfeeding. From complementary data, mothers did it in various rooms – in toilets, hallways, in the car. Half of the breastfeeding women outside the home were not satisfied with the conditions in which they placed their babies on the breast. Every tenth mother admitted having been openly criticised for breastfeeding in a public place.

Nevertheless, about 90% of them admitted that they put their baby to the breast outside the home (Table 2).

**Table 2:** Survey results

Questions and Answers	N (%) *
What do you think about breastfeeding in public places (gallery, train station, park, office)?	
I can	634 (90,6)
I have no opinion	38 (5,4)
I shouldn’t	28 (4,0)

Have you ever breastfed in a public place?	
Yes	547 (78,9)
No **	147 (21,1)

If Yes, how often did you breastfeed in a public place?	
Occasionally	178 (30,8)
Sometimes	195 (33,8)
Often	147 (25,5)
Whenever	57 (9,9)

If Yes, was the place where you breastfed specially adapted to this purpose?	
Yes	45 (7,8)
No	393 (68,0)
Partially	140 (24,2)

If Yes, did you feel comfortable in the place where you breastfed?	
Yes	233 (46,9)
No	264 (53,1)

Have you ever been criticised for breastfeeding in public?	
Yes	66 (10,4)
No	568 (89,6)

Did you have to breastfeed outside your home?	
Yes ***	640 (91,4)
No	60 (8,6)

(* Missing data were case-wise deleted. ** ‘I had no such need’ 54,8%; ‘There were no suitable conditions for this’ 21,4%; ‘I was ashamed’ 9,7%; ‘It is not well perceived by other people’ 7,7%. *** ‘In my car’ 38,0%; ‘In the Mother and Child Room’ 23,6%; ‘In the lobby’ 19,5%; ‘In the toilet’ 4,8%.)

The frequency of breastfeeding a child in public was not statistically dependent on criticism from third parties (Table 3).

**Table 3:** Frequency of breastfeeding in a public place by the surveyed women by criticism phenomenon (p = 0,073)

If Yes, how often did you breastfeed in a public place?	Women criticised for public breastfeeding N (%)*	Women not criticised for public breastfeeding N (%)*
Occasionally	14 (21,9)	162 (31,8)
Sometimes	23 (35,9)	172 (33,7)
Often	17 (26,6)	129 (25,3)
Whenever	10 (15,6)	47 (9,2)

(* Missing data were case-wise deleted.)

Women in all types of localities are exposed to criticism of breastfeeding in public, with no particular preference (Table 4).

**Table 4:** Tolerance for seeing a woman breastfeeding in a public place by city size (p = 0,276)

Size of the city of residence (size of the population)	Women criticised for public breastfeeding N (%)*	Women not criticised for public breastfeeding N (%)*
Urban agglomeration (e.g. Warsaw, Katowice)	30 (13,2)	197 (86,8)
Large city (over 100 000)	11 (8,9)	112 (91,1)
Average city (between 20 000 – 100 000)	13 (7,2)	168 (92,8)
Small city (< 20 000)	12 (11,8)	90 (88,2)

(* Missing data were case-wise deleted.)

## Discussion

Women who took part in the study want to breastfeed outside their homes and believe that there should be places for breastfeeding in public areas. More than three-quarters of them admitted breastfeeding outside the home. As shown by this anonymous diagnostic survey conducted among women in different ages, living in cities of various sizes and with different lactation experiences, many feel embarrassed to breastfeed in public. There are no premises for breastfeeding, and no social acceptance for the sight of a nursing mother. Avoidance of breastfeeding by women results both from embarrassment and fear of discomfort experienced by others and their negative evaluation. The lack of epidemiological safety and the child's discomfort are emphasised.

Similar results are also shown by other surveys and interviews [26], [30, 31, 32]. In the article ‘It's okay to breastfeed in public but...’ the controversy of the survey respondents concerns whether breastfeeding should be public at all. The respondents believe that when feeding in public, mothers should be discreet by choosing the right place to minimise the discomfort felt by others, and to protect themselves from judgement and unwanted glances [26].

Our respondents also repeatedly stated that they did not feel comfortable having their baby breastfed outside their homes. One in ten experienced direct unpleasant comments or even criticism while breastfeeding in public.

In European countries and America, mothers breastfeed their babies everywhere, but usually cover their breasts [27].

It is generally accepted by the environment and considered as common and ordinary activity [33, 34, 35].

The authors of the survey in Accra (Ghana) observed that, although some women experience discomfort with breastfeeding in public places, it is a common phenomenon. Most mothers cover their breasts when breastfeeding and feel that separate areas should be used [30].

In another international study, the authors aimed to understand the concerns of women who are breastfeeding in public. In Australia, Ireland and Sweden, respondents indicated that feelings of awkwardness and discomfort discourage public breastfeeding [31].

Mothers in Poland do not find a suitable place to breastfeeding. For this reason, many breastfed in isolation from their surroundings – in a car in a parking lot, on a park bench, and even in a toilet. Finally, many breastfeeding women, fearing the need to feed, gave up leaving home or taking their baby with them. Every fourth respondent sees a substitute for breastfeeding conditions, and only less than 8% confirm the existence of a professional place for breastfeeding.

In a two-stage study conducted in California, women in the second part of the study were asked about the so-called ‘Nurses' corners’, that is, separate places for breastfeeding. The best features of such places were indicated by the surveyed women: comfort (50%), privacy (19%), that they feel welcomed and supported (14%), that it is a good idea (13%) and convenience (6%) [32].

A survey in Ottawa (Canada) shows public support for public breastfeeding in a restaurant and mall, where 75% of respondents approved breastfeeding in public (78% in a restaurant, 81% in a shopping centres). People without children, with lower education, with a native language other than English or French, and retired people were not in favour of breastfeeding in public. The authors conclude that despite the favourable law, it is still necessary to improve the tolerance and understanding of breastfeeding in public [36].

Another study on the perception and acceptance of public breastfeeding was conducted in Germany. Six percent of mothers confirmed that they had experienced negative or very negative social reactions related to breastfeeding in public, and 80% of mothers and 66% of the general population believe that breastfeeding should be possible anytime, anywhere. However, just over half (54%) of the surveyed nursing mothers would dare to breastfeed in locations such as cafes and restaurants. Approval to breastfeed in places such as restaurant and cafes is given by less than half of the respondents from the general group (except for nursing mothers). The researchers emphasise that there is no specific target group that could improve the acceptance of breastfeeding in public. Thus, certain conflicts and situations in public were observed. Their identification can help in developing strategies to increase acceptance of the widespread use of public places for breastfeeding [37].

The authors of the study, in which physicians, residents and medical students participated, also observed that the barriers experienced by breastfeeding mothers are lack of adequate space at work and time constraints, and these barriers disrupt breastfeeding times, even for women working in medicine [25].

## Strengths and limitations

The social environment's perception of a mother who breastfeeds outside the home and the problems she experiences can become factors in her decision to continue or not to breastfeed.

Therefore, conducting research on this problem and publishing its results is a significant scientific evidence for promoting breastfeeding in the public space, identifying barriers related to it and formulating recommendations that can be the basis for introducing system changes and building tools for influencing the society.

We realise that the opinion of 700 respondents may not be sufficient to define such a common problem as breastfeeding in public. We feel a certain dissatisfaction due to the small number of responses from women from various backgrounds with a small population. Moreover, the selection of the group was random.

There have not been many studies done on groups of women who are breastfeeding or have breastfed in the past. The strengths of this study may be to present opinions of only nursing women (now or in the past) on this subject because their views can explain the reasons and outline a way to solve the problem of negative experiences related to breastfeeding in public. For this reason, we believe that the data from 700 questionnaires from nursing women are a strong asset of the study. Additionally, the population of the study was diverse in terms of size of the city of residence and age, and diverse in terms of education, although the differences were greater here (most with higher education).

The effect of the possibility of breastfeeding in a public place on the duration of breastfeeding has not been analysed. We believe that women's views on public breastfeeding in relation to the length of breastfeeding would be another interesting aspect of our further job.

## Conclusion

The results of our study indicate that most breastfeeding women do so in public when the need arises. They try to breastfeed discreetly and comfortably for themselves and the baby, as well as for those around them. Sometimes it is associated with looking for a secluded place and covering themselves, breastfeeding for example in the car or even in the toilet. The survey also shows that there is a group of women who do not breastfeed in public due to the lack of a suitable place, embarrassment and fear that it will not be perceived well by other people.

It is a strong signal to direct our actions towards creating special areas for breastfeeding in public (i.e., offices, shopping canters, restaurants, recreational facilities, public transport stations, etc.). The creation of separate mother and child-friendly spaces, ensuring comfort and discretion during breastfeeding, can help to minimise the discomfort of the mother, baby and other people around.

We are convinced that the need to create friendly places for breastfeeding fulfils the fundamental needs of a child..

If friendly places existed, then nursing women would be able to leave their homes and breastfeed in public without facing discrimination .

## Figures and Tables

**Figure 1:**
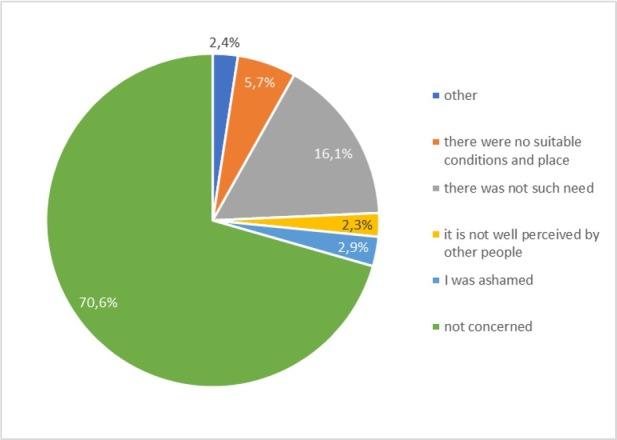
Reasons for abstaining from breastfeeding in public

**Figure 2:**
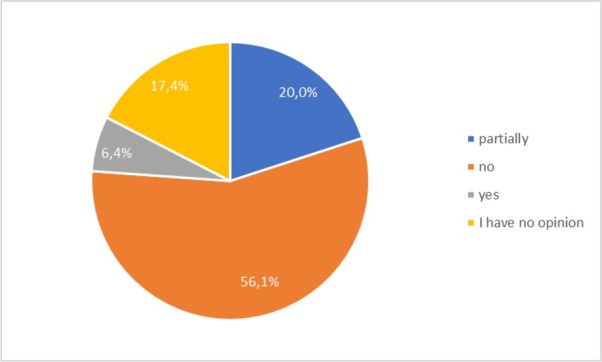
Adaptation of places for breastfeeding in the opinion of breastfeeding women

**Figure 3:**
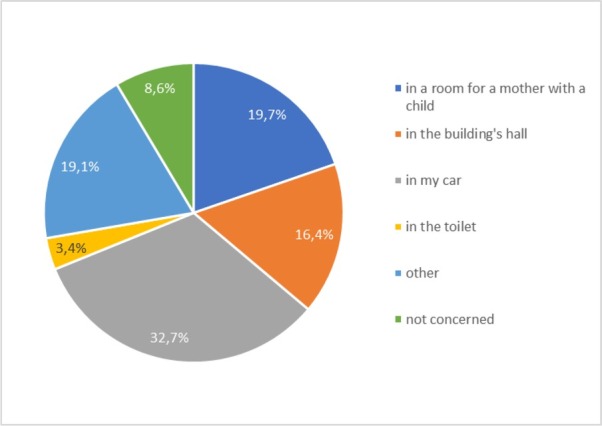
Places in public places where the respondents breastfed

**Figure 4:**
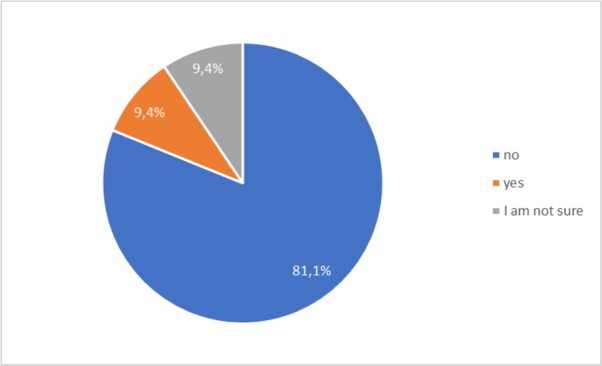
Criticism of breastfeeding a child in public

## Appendix

**Figure 1**. Reasons for abstaining from breastfeeding in public

**Figure 2**. Adaptation of places for breastfeeding in the opinion of breastfeeding women

**Figure 3**. Places in public places where the respondents breastfed

**Figure 4**. Criticism of breastfeeding a child in public
